# A Case Report of Angioedema and Anaphylactic Shock Induced by Ingestion of Polyethylene Glycol

**DOI:** 10.5811/cpcem.2020.3.45218

**Published:** 2020-04-23

**Authors:** Amy Rossi, Lesley Osborn

**Affiliations:** University of Texas at Houston, McGovern Medical School, Department of Emergency Medicine, Houston, Texas

**Keywords:** Polyethylene glycol, PEG, allergic, angioedema

## Abstract

**Introduction:**

We report one of few documented cases of a severe anaphylactic reaction with angioedema to polyethylene glycol (PEG).

**Case Report:**

The patient presented 30 minutes after onset of his symptoms and quickly developed hypoxia and hypotension refractory to intramuscular epinephrine, intravenous fluids, methylprednisolone, and supplemental oxygen via non-rebreather mask. He ultimately required intubation, an epinephrine infusion, and admission to the medical intensive care unit.

**Discussion:**

This case depicts a clinical reaction to PEG, a medication rarely implicated in severe anaphylaxis or angioedema.

**Conclusion:**

The allergenic potential of PEG-containing products should be raised, and providers should have a heightened awareness of these potential side effects.

## INTRODUCTION

Polyethylene glycol (PEG) is a compound found in a variety of products classically viewed as chemically inert substances.^1^ In the medical community, it is recognized most frequently as the active ingredient in bowel-cleansing regimens such as Miralax, Go-Lytely, or Half-Lytely. It is also an additive in numerous other medications and substances ranging from intramuscular (IM) depo-provera to antibiotic tablets and ultrasound gel.^2^ Upon review of the literature, we present only the second published case of a severe allergic reaction involving both anaphylactic shock and angioedema to PEG, particularly unique in its severity despite typical anaphylaxis treatment. Fourteen cases of anaphylactic shock secondary to substances containing PEG have been reported; however, only one of these was further complicated by angioedema.^3^,^4^ In the emergency department (ED), this additional complication can drastically change management of patients, necessitating an awareness of the severity of this reaction to this commonly prescribed medication.

## CASE REPORT

A 76-year-old obese Caucasian male with past medical history of hypertension controlled with hydrochlorothiazide, chronic obstructive pulmonary disease on ipratropium bromide and albuterol, diabetes mellitus type II on metformin, atrial fibrillation on diltiazem and warfarin, gastroesophageal reflux disorder, and benign prostatic hypertrophy on tamsulosin presented to the ED with acute-onset shortness of breath. On initial examination, the patient was in respiratory distress, unable to speak greater than two-word phrases, with diffuse erythema and associated severe pruritus. He described an acute onset of these symptoms approximately 30 minutes prior to arrival to the ED. On initial evaluation, he gave us a piece of paper on which he had written “polyethylene glycol,” implicating this as the new and only medication or substance he had ingested in the three hours prior to presentation.

At the time of arrival, the patient’s blood pressure (BP) was 177/143 millimeters of mercury (mmHg), heart rate (HR) 163 beats per minute (bpm), respiratory rate (RR) 23 breaths per minute, oxygen saturation of 93% on room air, weight 103 kilograms (kg). He was in acute respiratory distress in tripod position, with an urticarial eruption on his trunk. Auscultation was significant for inspiratory and expiratory wheezes in all lung fields. Oropharyngeal exam revealed an edematous soft palate with a brawny texture and elevation of his tongue to the hard palate, with associated difficulty tolerating his oral secretions.

The patient immediately received 0.3 milligrams (mg) of IM epinephrine, 125 mg intravenous (IV) methylprednisolone, 50 mg IV diphenhydramine, 20 mg IV famotidine, and one liter 0.9% normal saline. Despite these interventions, the patient deteriorated rapidly, demonstrating signs of anaphylactic shock. His BP decreased to 63/42 mmHg, with a HR of 173 bpm, RR of 34 breaths per minute, and oxygen saturation of 78% on room air. It was determined that the patient would need intubation and further resuscitation with IV fluids and an epinephrine infusion to maintain his blood pressure in the setting of respiratory failure and anaphylactic shock. His oxygenation improved to 93% with application of supplemental oxygen via a non-rebreather mask at 15 liters per minute, and he maintained his mentation; thus, we elected to use fiberoptic nasopharyngoscopy to evaluate the airway for concern of significant oropharyngeal edema and the potential for a complicated endotracheal intubation.

The patient’s oropharynx was prepped with topical benzocaine spray, and a 6.0 endotracheal tube (ETT) was loaded on the fiberoptic scope in preparation for emergent nasotracheal intubation if necessary during the procedure. On visualization of the pharynx, he was noted to have edema extending to the area of the hypopharynx, sparing the epiglottis and vocal cords. We elected to proceed with rapid sequence endotracheal intubation after evaluation of his airway. The intubation was performed with 200 mg IV ketamine and 200 mg IV succinylcholine. Video laryngoscopy using a 7.0 ETT resulted in first-pass success. An in-line albuterol nebulizer was then initiated. To allow for central hemodynamic monitoring and accurate titration of the patient’s epinephrine infusion, right femoral central and arterial lines were placed with goal to maintain a mean arterial pressure of greater than 65 mmHg. The patient was subsequently admitted to the medical intensive care unit in critical condition.

## DISCUSSION

PEGs are polymers composed of ethylene oxide that are non-ionic and hydrophilic and are thought to be chemically inert.^1^ They are commonly used in industry production of medications, medical products, and cosmetics.^1^ Given its chemical structure as a large hydrophilic polymer, it is effective as a bowel regimen as it does not readily cross the mucosal surface of the gastrointestinal tract.^4^,^6^ Due to this inherent quality, the compound has generally been viewed as minimally antigenic or reactive.^6^ However, larger PEG polymers (>1000 atomic mass units) have immunogenic properties as they are likely large enough to elicit immune responses.^6^ While in general these are poorly absorbed, it has been demonstrated that PEG polymers can be recovered in the urine and would, therefore, be able to elicit immune responses in these subjects.^7^

**CPC-EM Capsule**

What do we already know about this clinical entity?

Histaminergic angioedema and anaphylaxis are similar disease processes, commonly seen in the emergency department in various states of severity.

What makes this presentation of disease reportable?

Polyethylene glycol (PEG) is thought to be minimally immunogenic; however, this case report shows it may have severe allergic potential in some patients.

What is the major learning point?

All medications should be considered in an anaphylactic reaction.

How might this improve emergency medicine practice?

Emergency physicians may more readily anticipate severe reactions and a difficult airway if patients present with signs and symptoms of allergy to products containing PEG.

Of those cases with reported reactions to PEG-containing products, the majority of patients present with simple urticaria or meet criteria of anaphylaxis with greater than two body systems involved.^3^,^8^ However, more severe reactions involving anaphylactic shock and occurrences of ventricular arrhythmias have been documented.^3^,^9^ Wenande et al recently reported a summary of the known cases of allergic reactions to PEG-containing products.^3^ Of those reported, fewer than 20 patients experienced cardiovascular collapse indicative of anaphylactic shock and only one with co-existing angioedema.^3^ All cases reported with discussion of patient management detailed resolution of anaphylactic symptoms and angioedema over the course of hours with recurrent dosing of epinephrine, antihistamines, and steroids.^3^

Our case differed in severity to those cited above, as the patient’s quick decompensation necessitated an escalation in treatment. The primary change in management was introduction of continuous IV epinephrine to support BP. Current guidelines support administration of IM epinephrine to stable patients demonstrating anaphylaxis; however, they recommend administration of IV epinephrine if anaphylaxis appears to be severe with an immediate life-threatening manifestation. The recommended starting infusion rate is between 1–4 micrograms per minute.^10^ Other treatment modalities remained the same, including steroid dosing and use of H1 (histamine type 1) and H2 antagonists.

The case reported here also presented a potentially difficult endotracheal intubation secondary to the significant angioedema visualized on his initial bedside oropharyngeal exam. It was presumed to be resultant of a histaminergic reaction given the concurrent anaphylaxis, as opposed to a non-histaminergic cause, such as bradykinin-mediated or hereditary angioedema, which would require a different treatment regimen.^11^ The patient was not actively taking angiotensin-converting-enzyme inhibitors for hypertension, one of the most implicated substances in bradykinin-mediated angioedema.^12^ He had not previously had an allergic reaction or edema of his lips or oropharynx to any other substance per his report. He also had no personal or family history of similar presentations, which makes it unlikely that he suffers from hereditary angioedema.^12^

Histaminergic angioedema is a subtype of angioedema caused by a deep tissue reaction initiated by histamine release from mast cells, and by subsequent immunoglobulin E-mediated complement activation.^2^ It causes vasodilation and vascular permeability, typically affecting the perioral and periorbital regions, but can also be seen as a non-pitting edema of the extremities and abdomen.^5^ When this reaction localizes to the oropharyngeal structures, endotracheal intubation may become extremely challenging due to severe hypopharyngeal and vocal cord edema.

Studies vary on prevalence of airway obstruction with angioedema; however, in general about 15% of all types of angioedema cases require intubation, and 50% of those needing a definitive airway require cricothyrotomy or tracheostomy.^12^ We identified no recent studies with a PubMed search on rates of intubation in histamine-mediated angioedema specifically. Histaminergic angioedema is mediated by the cytokine and histamine activation implicated in anaphylaxis, and the use of antihistamines, steroids, and epinephrine is regarded as the standard of care.^12^ Treatment regimens for bradykinin-induced and hereditary angioedema continue to be controversial.^2^,^11^ Regardless of the cause, ensuring establishment of a definitive airway, when needed, is paramount.

## CONCLUSION

The exact mechanism of PEG-induced anaphylaxis has not yet been fully elucidated. It is likely initiated by uptake of PEG molecules across the gastrointestinal mucosa, and its large molecular weight and hydrophilicity prolongs this process. We suspect that the patient was therefore able to tolerate a significant ingestion prior to onset of symptoms, and the amount ingested was likely the cause of his rapid deterioration to anaphylactic shock, despite the appropriate initial management for anaphylaxis. Although the associated side effects of PEGs are commonly restricted to abdominal discomfort, bloating, cramping, and nausea, case reports like this one suggest a need for increased awareness in all specialties that regularly prescribe these medications. Emergency providers should be especially aware of this reported complication of PEG-containing products as rapid identification and resuscitation is key to improving morbidity and mortality in these patients.

## References

[1] Shah S, Prematta T, Adkinson NF (2013). Hypersensitivity to polyethylene glycols. J Clin Pharmacol.

[2] Wilkerson R (2012). Angioedema in the Emergency department: an evidenced-based review. Emerg Med Pract.

[3] Wenande E, Garvey LH (2016). Immediate-type hypersensitivity to polyethylene glycols: a review. Clin Exp Allergy.

[4] Gachoka D (2015). Polyethylene glycol (PEG)-induced anaphylactic reaction during bowel preparation. ACG Case Rep J.

[5] Lewis LM (2013). Angioedema: etiology, pathophysiology, current and emerging therapies. J Emerg Med.

[6] Knop K, Hoogenboom R, Fischer D (2010). Poly(ethylene glycol) in drug delivery: pros and cons as well as potential alternatives. Angew Chem Int Ed Engl.

[7] Brady CE, DiPalma JA, Morawski SG (1986). Urinary excretion of polyethylene glycol 3350 and sulfate after gut lavage with a polyethylene glycol electrolyte lavage solution. Gastroenterology.

[8] Sampson HA, Muñoz-Furlong A, Campbell RL (2006). Second symposium on the definition and management of anaphylaxis: summary report--Second National Institute of Allergy and Infectious Disease/Food Allergy and Anaphylaxis Network Symposium. J Allergy Clin Immunol.

[9] Marsh WM, Bronner MH, Yantis PL (1986). Ventricular ectopy associated with perioral colonic lavage. Gastrointest Endosc.

[10] ECC Committee and Subcommittees and Task Forces of the American Heart Association (2005). American Heart Association Guidelines for Cardiopulmonary Resuscitation and Emergency Cardiovascular Care. Circulation.

[11] Long BJ, Koyfman A, Gottlieb M (2019). Evaluation and management of angioedema in the emergency department. West J Emerg Med.

[12] Depetri F, Tedeschi A, Cugno M (2019). Angioedema and emergency medicine: from pathophysiology to diagnosis and treatment. Eur J Intern Med.

